# Can Repeated Reductive Genome Evolution Across Archaeplastida Explain Volatility in Traits?

**DOI:** 10.1002/ggn2.70048

**Published:** 2026-10-24

**Authors:** Cäcilia F. Kunz, Merve Nida Baştürk, Jan de Vries, Hengchi Chen

**Affiliations:** ^1^ Institute of Microbiology and Genetics Department of Applied Bioinformatics University of Göttingen Göttingen Germany; ^2^ Göttingen Centre for Molecular Biosciences (GZMB) University of Göttingen Department of Applied Bioinformatics Göttingen Germany; ^3^ Campus Institute Data Science (CIDAS) Department of Applied Bioinformatics University of Göttingen Göttingen Germany

**Keywords:** biology, dna sequencing, evolutionary biology, genome evolution, genome, plastid, trait

## Abstract

Genome sequencing efforts have covered phylodiverse Archaeplastida, the lineage of plants and algae that bears primary plastids. In these efforts, gene gains and novelties are a major focus of comparative genomic analyses. Yet, ancestral reconstructions increasingly infer extensive gene loss in major lineages, challenging narratives that emphasize continual gains in complexity. In this perspective, we examine evidence for recurrent genome contraction across photosynthetic eukaryotes, focusing on red algae, the ancestry of land plants, and subsequent diversification within embryophytes. Recent studies suggest that ancestors of modern lineages often possessed larger and more complex gene repertoires than their descendants. We argue that repeated cycles of genome expansion and reduction have generated substantial trait volatility, making gene loss a central driver of eukaryotic evolutionary innovation and diversification.

## Graphical abstract

Genome evolution in Archaeplastida is often framed around gene gains and increasing complexity. Yet ancestral reconstructions reveal recurrent genome contraction, with ancestors frequently harboring larger gene repertoires than descendants. Across red algae, land‐plant ancestry, and embryophyte diversification, repeated expansion and reduction cycles generated trait volatility, positioning gene loss as a driver of innovation.

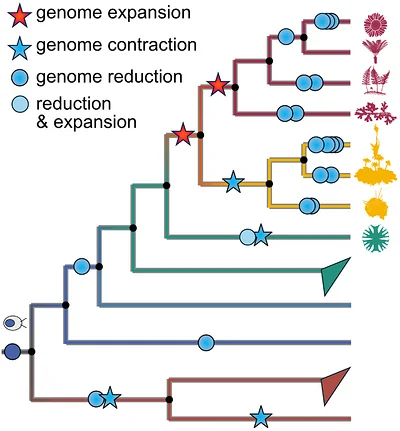

## 1 Introduction

Genome reduction is a force well‐recognized in the evolution of prokaryotes, and upon lifestyle changes toward obligate dependencies such as seen in endosymbionts and parasites [1, 2] (Figure 1). Yet it is a general force in the evolution of genomes of any eukaryote.

**FIGURE 1:**
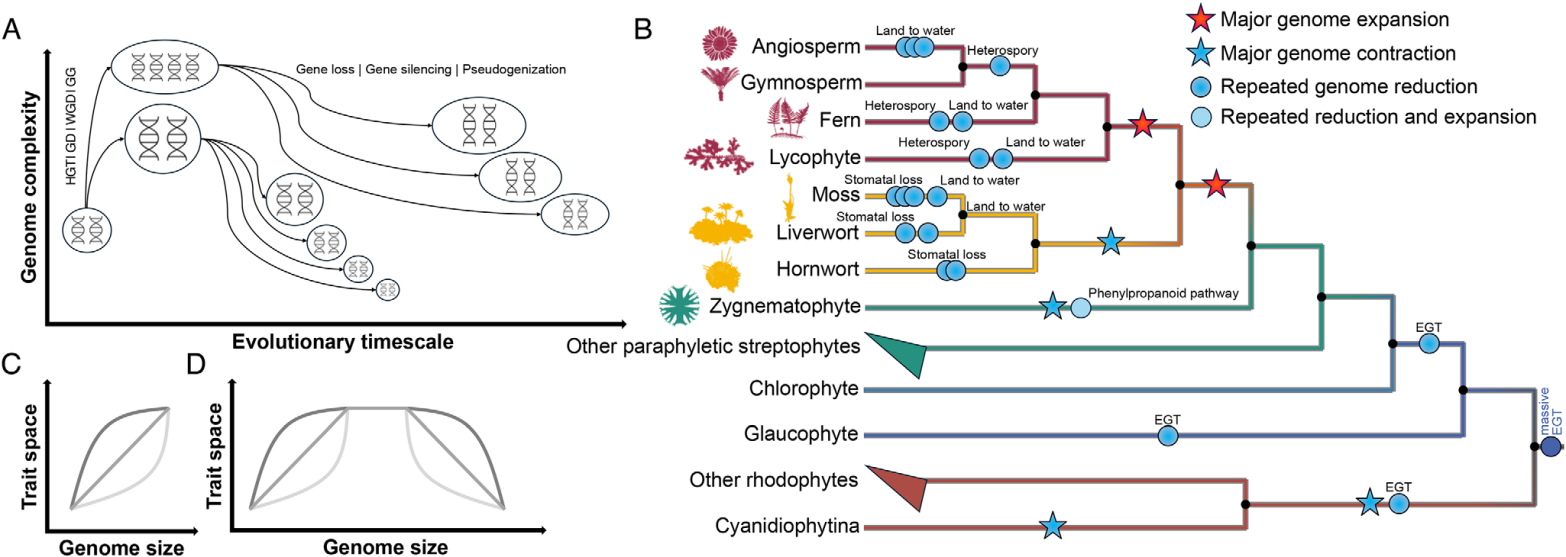
Expansive and reductive genome evolution. (A) a schematic representation of genome expansion through horizontal gene transfer (HGT), gene duplication (GD), whole‐genome duplication (WGD), gene gain (GG), and of genome reduction through gene loss, gene silencing, pseudogenization. (B) a cladogram of Archaeplastida showing the major episodes of genome expansion, genome reduction, repeated genome reduction, and repeated reduction and expansion discussed in this study. (C) a hypothetic relationship between genome size and trait space when the genetic repertoire is relatively small, in which differently shaded curves represent alternative relationships with different curvatures. (D) extending the hypothetic relationship between genome size and trait space in C to scenarios where the genetic repertoire is relatively larger such that there are multiple genetic routes to realize the same phenotype and larger genome size may have lower fitness and thus less viable trait space under environment‐specific selection. The silhouettes of the angiosperm, gymnosperm, fern, lycophyte, moss, liverwort, hornwort, and zygnematophyte are represented by the species of *Helianthus annuus*, *Cycas circinalis*, *Polypodium vulgare*, *Selaginella kraussiana*, *Physcomitrium pyriforme*, *Marchantia polymorpha*, *Anthoceros agrestis*, *Micrasterias radiata*, respectively. These silhouettes are distributed in PhyloPic under the CC0 1.0 Universal Public Domain Dedication license.

Across the diversity of eukaryotes continuously more genomes are being sequenced [3]. And thus, we find more and more genes that are unique to certain lineages. If we impose a scenario where we combine the assumptions that (i) most genes are born out of other, existing genes and (ii) most genes are inherited vertically, we might end up with something that has in other contexts been coined the “Genome of Eden” problem for last common ancestors [4]. In brief, the “Genome of Eden” refers to a scenario in genome evolutionary biology where a hypothetical, all‐encompassing ancestral organism possessed every gene currently found in its descendants; it highlights the implausibility of assuming that all gene variations are due solely to gene genesis and loss, rather than lateral gene transfer. In fact, extant eukaryote genomes document substantial episodes of gene gain, duplication, transfer, and loss [5, 6, 7, 8, 9]. While the evolutionary significance of acquiring extra genes is readily envisionable in that it expands the gene repertoire and increases complexity, the adaptive edge of gene loss—despite its repeated occurrences coinciding with major life transitions (Figure 1B)—is much less appreciated.

With an ever‐increasing sampling of eukaryote genomes, we infer throughout evolutionary history massive gene family and genome contraction for major lineages. Archaeplastida are photosynthetic eukaryotes with primary endosymbiosis‐derived plastids [10]. The evolution from ancestral to extant Archaeplastida walked through repeated yet divergent reductive evolutionary paths through massive endosymbiotic gene transfer (EGT) episodes [11, 12, 13] (Figure 1B), transforming the ancestral cyanobacterial endosymbiont with several thousand genes into  distinct plastids retaining only hundreds of genes or less [14] and ultimately ushering in the immense genomic and phenotypic diversity of primary plastid‐bearing algae and plants, including land plants (embryophytes). Leveraging probabilistic phylogenomic approaches, Harris et al. [15]. reconstructed ancestral gene content and found that the origin of land plants was marked by substantial gene‐family expansion, driven primarily by gene duplications, suggesting that the common ancestor of embryophytes and Zygnematophyceae already possessed many of the genetic components associated with plant complexity; in contrast, both the bryophyte and tracheophyte lineages experienced major genomic restructuring after diverging from this ancestor (Figure 1B), with bryophytes showing especially extensive and robustly supported gene loss—including genes associated with shoot and root development—suggesting that modern bryophytes are evolutionarily reduced relative to the ancestral embryophyte. Opposite the axis of terrestrialization, the secondary transition from land back to water has repeatedly occurred at least 132 times (Figure 1B) and ushered in a variety of aquatic embryophyte lineages that have undergone consistent genomic reduction whose extent can be well predicted by the gradient of their aquatic life forms (i.e., emergent, floating‐leaved, free‐floating, or submerged); the function of those convergently contracted gene families thereof is enriched in body plan organization, drought response, hormone regulation, and microbial defense [16], reflecting the adaptive fluidity of reductive evolution to the shift of selective regimes in the aquatic environment.

Archaeplastida are not the only lineage where reductive genome evolution has been documented at scale. Across 102 animal genomes, Guijarro‐Clarke et al. [17]. found extensive gene loss in Ecdysozoa and Deuterostomia, and the largest reductions of gene complement, alongside gene novelty, in flatworms, nematodes and tardigrades. They concluded that reductive evolution at the protein‐coding level shaped animal genome composition. In fungi and their relatives, Merényi et al. [18]. showed that ancestral protist gene families were lost gradually and step by step. These losses ran parallel to episodic bursts of duplication and the progressive appearance of novel gene families, which the authors linked to key fungal traits such as osmotrophy and hyphal growth.

Loss and gain co‐occur—also and in Archaeplastida, where genome reduction is further coupled across compartments through endosymbiotic gene transfer. Loss from the plastid genome is often relocation to the nucleus rather than erasure, which makes “loss” a less binary category than in animals or fungi. Further, Archaeplastida have gone back and forth between major environments: from water to land, and then back to water at a minimum of the aforementioned 132 times. This offers a natural setting to ask whether reduction is reversible and predictable, rather than a one‐way sequence of events. By “trait volatility” we mean more than the known pattern of gene pools being shaped by selection toward particular environments. We propose that ancestral loss channels subsequent evolution. A trait that returns after a reduction is rarely rebuilt from the same gene complement. Instead, it is reassembled from retained remnants and lineage‐specific expansions, often through different genetic components (see the phenylpropanoid pathway, short PPP, below). This leads to a testable prediction: after an ancestral reduction, functional outputs should be recovered more often than their original genetic basis. Genomic comparison combined with biochemical and physiological data can test this.

Here, we explore how these dynamic forces boil down genomes of major lineages of Archaeplastida, undergoing extensive gene loss after periods of innovation. Such reductions can erase developmental and metabolic capacities, making extant taxa appear simplified relative to their ancestors—while lingering genetic and genomic potential remains. And after reductions, periods of innovations and lineage‐specific expansions can happen again. We propose that trait volatility in eukaryotes often reflects recurrent shrinkage and expansion of coding capacity across deep time.

### 1.1 Ancient Reduction in Red Algae

Rhodophytes (red algae) are an interesting case for our discussion: They show phases of massive reduction of their gene inventories at the phylum level that resulted in the loss of many functions conserved in free‐living eukaryotes (Figure 1B). Yet, rhodophytes, remain a species‐rich and diverse lineage in terms of morphology and habitats and exhibit complex reproductive strategies.

Rhodophyta are one of the three main lineages of Archaeplastida [10] (Figure 1B). Together with the nonphotosynthetic lineages Rhodelphidia and Picozoa they constitute Rhodaria, which is sister to the monophyly of Chloroplastida and Glaucophyta [10, 19]. According to the fossil record, rhodophytes have existed for at least 1.03 billion years [20] (Ga). There are around 7,700 described extant species divided into two subdivisions [21]: Cyanidiophytina (unicellular thermophilic red algae) and Rhodophytina (mesophilic red algae accounting for the vast majority of red algal diversity) [22, 23].

At least two major phases of gene loss have been inferred for the Rhodophyta [24] (Figure 1B). First, in the stem lineage before the split of Cyanidiophytina and Rhodophytina [24], lost roughly 25% of the ancestral gene inventory [13] and resulted in a reduced nuclear genome (5,000–14,000 genes) as compared to the gene number we can assume for the LCA of Chloroplastida [10]. Pathways and functions lost include flagellum‐based motility, pyrimidine degradation, sulfases, phytochrome‐based light sensing, glycosylphosphatidylinositol (GPI)‐anchor biosynthesis, and macroautophagy pathways, alongside a reduction in cytoskeletal complexity [13, 25]. Second, the ancestor of Cyanidiophytina [24], lost afurther genetic capacity, most notably the nickel‐dependent urease pathway [13].

What explains these reduction events? Obviously, this requires a different explanation than the extreme genome reduction seen in parasites and endosymbionts, since rhodophytes are free‐living with large populations [13]. The favoured adaptive account holds that the ancestor of Rhodophyta shifted into an oligotrophic or extreme niche where streamlining became favourable, a “less is more” logic [13, 26]. Neutral explanations offer a complementary mechanism for why loss spread through whole interdependent pathways rather than gene by gene: once relaxed selection allowed one pathway component to degrade, others depending on it could become easily dispensable, in turn, producing an iterative contingent, effectively irreversible cascade without requiring every gene to be independently favoured for loss [27]. Under this view, the second phase of loss reads as a continuation of the first, so the continued streamlining: the extremophilic lifestyle shaping the stem lineage was maintained and intensified in early Cyanidiophytina, adding hot‐spring‐specific losses on top of the ancestral reduction, while Rhodophytina diverged toward milder habitats [13]. Separate from this gene‐inventory story is the genome‐size growth seen in some red seaweeds via transposable element expansion, better explained by the drift‐barrier hypothesis, where smaller effective population sizes weaken selection against mildly deleterious insertions, than by adaptation [28, 29].

Rhodophytina, having evolved from an ancestor that lost many essential eukaryotic traits and associated gene inventory, radiated into a species‐rich lineage ranging from unicellular to multicellular. Indeed, they might be the oldest case of eukaryotic mulitcellularity [20, 30]. Rhodophytina occupy diverse habitats and showing a mix of reproductive strategies, like an elaborate triphasic sexual life cycle unusual among eukaryotes [25, 31, 32]. Looking at the genomes of these algae, we learned the following: mesophilic red algae diversification is not explained by reacquisition of the ancestral gene inventory, but by secondary, lineage specific gene family expansions layered onto the reduced genome [33, 34]. Phycobilisome linker proteins illustrate this: an ancestral linker gene reached the nucleus via endosymbiotic gene transfer during primary endosymbiosis, and additional linker families later diversified specifically in mesophilic lineages, expanding light‐harvesting capacity across diverse light environments, while Cyanidiophytina instead lost phycoerythrin and simplified its light‐harvesting structures [33]. Transcription factor complements show a similar pattern: total family size does not differ between unicellular and multicellular red algae, but specific families do: C2H2 zinc‐finger genes are expanded in multicellular Florideophyceae and Bangiophyceae relative to Cyanidiales [34]. Complex reproduction follows the same pattern: in *Bostrychia*, gene‐rich UV sex chromosomes carry a sex‐linked TALE‐HD transcription factor orthologous to regulators of haploid–diploid transitions in other multicellular lineages, tying the triphasic life cycle to lineage‐specific genetic novelty rather than ancestral inheritance [35]. Another aspect is genetic innovation. That the lost functions are not reacquired might indicate the possibility of the evolution of novel regulatory pathways, alternate functional pathways and protein multitasking, as proposed previously [13].

One important aspect of reductive nuclear genome evolution is its implications on the plastid genome evolution. Rhodophytes have gene‐rich plastid genomes compared to the other photosynthetic eukaryotes and it is hypothesized that successful EGT and loss of organelle copies of transferred genes is limited in rhodophytes due to the high rates of nuclear gene loss [36].

Taken together, Rhodophyta is a concrete case of trait volatility: one ancestral reduction event followed by lineage‐specific divergence, in which one branch continued the reductive trajectory while another left it behind, repeatedly generating new complexity through independent genetic means rather than a shared trajectory.

### 1.2 A Streamlined Last Common Ancestor of Land Plants and Zygnematophytes

In the last 15 years, a rapidly increasing number of phylogenomic and transcriptomic analyses of both the nuclear and chloroplast genome has revealed that the closest relatives of land plants are the zygnematophytes [37, 38, 39]. This was not an expected finding. Of the three algal Phragmoplastophyta lineages that have been proposed as closest algal relatives over the years, the zygnematophytes are the least morphologically complex, being unicells or unbranching filaments (albeit branching occurs in some lineages). Now we know, owing to a combination of within‐class phylogenomics and ancestral character state reconstruction, that multicellular growth as well as increasing complexity in multicellular body plans has emerged multiple times independently in the streptophyte algal paraphylum [40, 41, 42, 43, 44, 45]. Indeed, the last common ancestor of zygnematophytes can be inferred as a unicell—with high probability [44] and multicellular growth could be trait with environment‐dependent volatility [41, 45, 46].

Morphological complexity does not necessarily correlate to genome size (Figures 1B–D). The genomes of many zygnematophytes are indeed extremely small—surprisingly so the filamentous species *Zygnema* *circumcarinatum* [47] (67.3–71.3 Mb) and *Spirogyra* *pratensis* [48] (55.44 Mb). In parallel, massive genomes with vastly expanded coding capacity also occur in zygnematophytes, exemplified by the large genome of the single‐celled *Penium* *margaritaceum* [49] (3.661 Gb).

To infer the biology of the last common ancestor (LCA) of land plants and its closest algal relative, we can take a phylogenetically informed look at the LCAs of zygnematophytes and embryophytes individually. Coming from the LCA of both (LCAEZ), there was likely a massive reduction in zygnematophytes on the one hand, while on the trajectory toward the LCA of embryophytes a huge jump in gene families occurred—both with regard to their innovation and expansion [15]. This could also be described as the trajectory between the first common ancestor (FCA) of either zygnematophytes or embryophytes, which in each case occurred as the first step in either direction from the LCAEZ.

Hence, the LCAEZ was more complex than the LCA of zygnematophytes, while having been less complex than the LCA of embryophytes. Coming back to the putative biology of the LCAEZ, how could it have looked like? What was the core that was necessary to be retained in zygnematophytes and could be expanded upon in embryophytes? What was dispensable, what essential? Even if a gene associated with a specific trait was dispensable in a given environmental niche, once it was lost it will inevitably have limited the ability of these species to radiate (back) into other environments. Yet, streptophyte algal evolution is rich in repeated habitat shifts [41, 42, 50, 51]. The (re‐)adaptation to another environment was thus likely ushered in by the recruitment of different genes necessary for the lifestyle in that habitat—a case of convergent evolution. Another facilitator could be horizontal gene transfer [52] (HGT). A similar case can be made for the multiple transitions in types of body plans [45]. These resulting nonhomologous genes required for a trait would then present an example of convergent evolution such as for the air pores in liverworts, as stomata are absent [53] (Figure 1B). Conversely, if a gene was not yet lost but pseudogenised, a reversal and putative subsequent expansion could increase lineage specific diversity and complexity; duplication can provide raw material for increased functional complexity, whereas differential gene loss shapes the distinctiveness and diversity of individual lineages [54] (Figure 1A,B).

Such reductive evolution might have also occurred in the case of the PPP (Figure 2). This pathway is a key stress response pathway in land plants, especially important in vascular plants as it can lead to the production of lignin monomers [55, 56, 57]. Many homologs of the genes of involved enzymes might have been present in the LCA of Streptophyta [58]. If one were to take the union of all enzyme‐coding genes potentially acting in the phenylpropanoid pathway across streptophyte algae, one would be able to draw an almost complete pathway map as we know it from plant textbooks—an almost complete pathway at a deep node. Yet, these genes occur in a patchy pattern along the leaves of the tree, with none of the leaves having the genetic foundation for the bona fide pathway. As such, the PPP stands as an example for the “Genome of Eden” problem. Lineage specific gene family expansions of, for example, cinnamyl alcohol dehydrogenase (CAD), occurred in both Chlorokybophyceae and Coleochaetophyceae. In zygnematophytes, in addition to the expansion, CAD split into two subfamilies, which are likely independent of the subfamilies of CAD found in embryophytes. However, in Charophyceae, CAD has been lost. Yet, the first committed step into the PPP is catalysed by phenylalanine ammonia lyase (PAL), of which there are only homologs identified in Charophyceae and Klebsormidiophyceae and no other streptophyte algae seem to possess PAL homologs [59, 60, 61]. These two examples showcase repeated loss and expansion of the involved genes (Figures 1A, B and Figure 2). In addition to this variation of presence/absence of homologs (and their expansions), even if present, the transcriptomic response might differ. In land plants, the PPP is activated upon UV‐B [62, 63, 64]. In a recently investigated zygnematophyte species, none of the canonical PPP enzymes were significantly differentially regulated after 2 h of UV‐B [65]. This is despite the production of diverse phenolic compounds in that zygnematophyte alga [65] (*Mesotaenium endlicherianum* SAG 12.97) and other zygnematophytes—where however also divergent routes likely operate [65, 66, 67, 68, 69, 70, 71].

**FIGURE 2:**
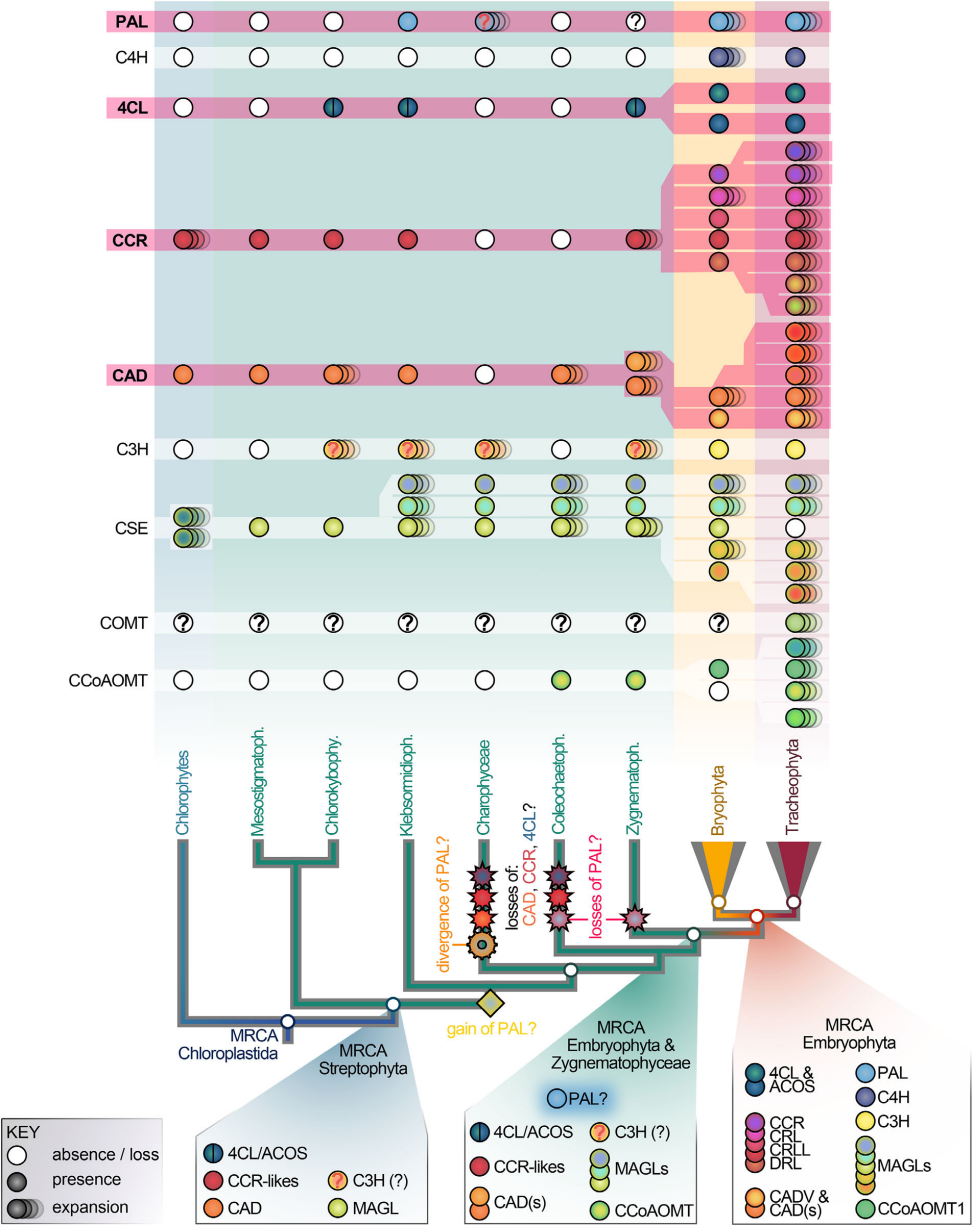
An example of the “Genome of Eden” problem: The phenylpropanoid pathway could be drawn almost completely at a deep node based on the union of all enzyme‐coding genes across phylodiverse streptophytes. Four evolutionary trajectories for enzyme coding genes with a pink background: in a gain only scenario, PAL might have been gained (yellow rhombus) in the LCA of K+ZCC+E, where it could have diverged (Charophyceae; cogwheel) and lost (Coleochaetophyceae, Zygnematophyceae; star); there is accumulating evidence suggesting that PAL was gained by horizontal gene transfer [41, 58, 59]. 4CL and CCR‐likes were likely present in the MRCA of Streptophyta and lost in Charophyceae and Coleochaetophyceae. CAD was likely present in the MRCA of Chloroplastida, lost in Charophyceae and expanded in zygnematophytes. Abbreviations: Phenylalanine ammonialyase (PAL), cinnamate 4‐hydroxylase (C4H), 4‐coumarate‐CoA ligase (4CL), cinnamoyl‐CoA reductase (CCR), cinnamyl alcohol dehydrogenase (CAD), 4‐coumarate 3‐hydroxylase (C3H), caffeoyl‐5‐Oshikimate esterase (CSE)/monoacylglycerol lipase (MAGL), caffeate O‐methyltransferase (COMT), and caffeoyl‐CoA O‐methyltransferase (CCoAOMT). Modified and based on de Vries et al. [58]. with permission.

The PPP thus bears upon volatility in a complex fashion. What we can document across streptophyte algae is volatility of the genetic components: whether the gene families encoding (putative) PPP enzymes are present, how many copies there are, and how they split into subfamilies. This is not the same as volatility of the functional traits, meaning the production of particular phenolic metabolites or the pathway's transcriptional response to UV‐B. Indeed, the several of the compounds are present—albeit it is neither from the same source nor the exact same compounds [65, 66, 68, 72, 73]. Gene‐family composition can change while metabolic output stays the same. For example, the same class of products may be made by non‐homologous enzymes or through alternative routes, as is likely the case in zygnematophytes [65, 66, 67, 68, 69, 70, 71]. In this scenario, similar to developmental system drift, the phenotype is conserved while its genetic basis turns over. The reverse also holds: conserved gene content does not guarantee conserved function. Canonical PPP genes in *M. endlicherianum* did not respond to UV‐B, yet this alga still produces diverse phenolic compounds [65]. The patchy distribution of PPP homologs should therefore not be read as repeated loss and regain of phenylpropanoid metabolism itself. That conclusion would require showing that some lineages lack the metabolic output and that others re‐evolved it independently. With the data now available, we treat the PPP as an example of volatility in the genetic toolkit. How this relates to the volatility of the metabolic and regulatory traits built on it remains to be tested biochemically and physiologically.

### 1.3 Genome Reduction Across Land Plants

The transition from water to land has repeatedly occurred across the tree of Archaeplastida [74]. However, only land plants (embryophytes) rose above their substrate and became the major extant terrestrial lineage of Archaeplastida that has achieved unparallelled ecological and evolutionary success on land [10, 75, 76]. Emerging from ancestral freshwater streptophyte algae [77, 78], extant embryophytes are defined by several synapomorphies: alternating between a haploid gametophyte generation developed from the spore, and a diploid sporophyte generation developed from the embryo retained in the maternal archegonium [79], which is also why they are called embryophytes. The emergence of embryophytes ∼500 Ma was accompanied by a string of phenotypic innovations (e.g., cuticle [80], stomata [81]) that facilitated their adaptation to terrestrial stressors [82] and complexified their body plans [83]. Unsurprisingly, ancestral embryophytes are inferred with massive episodes of gene duplication from their FCA to their LCA [15]. Surprisingly, it is the gene duplication rather than the de novo gene gain that predominantly underpins their genome complexification [15, 84, 85], suggesting that major evolutionary transition can be borne out of revamping existing genes and modules. Building on the significant ancestral genome expansion, however, extant embryophytes have repeatedly taken the reductive path to address a variety of environmental challenges and developmental needs (Figure 1B).

Arguably, the most striking case of reductive evolution repeatedly occurring in embryophytes is the development of gametophytes [86]. Tracheophytes run a sporophyte‐dominant life cycle in which the gametophyte generation is smaller in size and less developmentally complex than the sporophyte generation, in contrast to bryophytes [87, 88]. Nonetheless, ancestral tracheophytes—which are inferred as homosporous [89, 90]—still bore isospores that can develop into independent gametophytes [91]. The repeated emergence of heterospory in pteridophytes (e.g., Selaginellales, Isoetales, Salviniales) and spermatophytes (Figure 1B) gave rise to highly reduced endosporic gametophytes with fewer sperm and jacket cells than bryophytes [90, 92], for instance, in *Isoetes*, the antheridium comprises only eight cells (i.e., four spermatogenous cells surrounded by four peripheral cells), constituting the most reduced antheridium in pteridophytes [86]. Accompanied, the genome size and chromosome number of heterosporous pteridophytes have been found remarkably smaller than the homosporous pteridophytes [93, 94], likely due to the asymmetric megasporogenesis present in heterosporous lineages [95, 96, 97] that may lead to non‐Mendelian meiotic drive [98]. As such, the reductive development of gametophytes appears to come at the cost of reduced genomes of both gametophytes and sporophytes, but it has surely laid the ground for the ensuing pivotal water‐independent reproductive innovations of seeds and flowers that have ultimately ushered in the majority of land flora diversity as we see today.

On the vegetative innovation side, the emergence of stomata is inferred as one of the most crucial innovations of ancestral embryophytes [81, 99]. Essentially, stomata are epidermal pores bordered by a pair of guard cells [100]. Stoma‐bearing embryophytes can sense the exogenous environmental signals (e.g., light, atmospheric CO_2_ concentration, humidity) and endogenous signals (e.g., abscisic acid, short ABA) to control the stomatal aperture and density so as to flexibly regulate water loss and CO_2_ uptake [101, 102]; it should be noted that this signaling system likely has deep evolutionary origins before the dawn of land plants [103, 104, 105, 106]. Nonetheless, stomata have undergone repeated loss throughout the tree of embryophytes (Figure 1B). In mosses, stomata have been repeatedly lost at least 63 times [107]. In hornworts, stomata have been lost at least 2 times [108]. All extant liverworts lack stomata, which is inferred as a secondary loss [81]. In tracheophytes, stomata have been repeatedly lost in aquatic [109], poikilohydric [110], or parasitic [111] lineages. These episodes of stomatal loss are recurrently accompanied with gene loss [81, 99]. For instance, in seagrasses, 11 out of 30 guard cell toolkit related gene families have been convergently and completely lost while 6 have been significantly contracted [109], whereas in the floating alismatid the degree of loss is lesser, reflecting the adaptive flexibility to cope with different gradients of environmental changes and exemplifying the “use it or lose it” strategy for reductive genome evolution. The stomatal developmental pathway in ancestral embryophytes is inferred to comprise three key paralogous bHLH transcription factors FAMA, SMF and SPCH/MUTE [81]; all extant bryophytes have lost the FAMA and SPCH/MUTE while SMF has been lost in most tracheophyte lineages [81], indicating that the ancestral gene redundancy can repeatedly boil down to analogous developmental building blocks following different paths.

On the genome structure axis, polyploidy, or whole‐genome duplication, a strong evolutionary force, has repeatedly occurred across the tree of embryophytes, leading to great genome size variation [39, 112, 113, 114, 115] (Figure 1C,D). The establishment of polyploids generally follows a diploidy‐polyploidy‐diploidy path in which the parental diploid genome undergoes duplication and forms a polyploid genome which will go through diploidization back to a diploidized paleopolyploid, restoring bivalent pairing and meiotic stability [116]. Such processes of initial genome duplication followed by a long‐term genome boiling‐down process to diploidy have repeatedly coincided with the radiative evolution of numerous major plant lineages (e.g., Orchidaceae, Asteraceae [117]), reinforcing the potent evolutionary potential of the expansion‐followed‐by‐reduction path. Recent studies have also recurrently shown that it is the post‐polyploid downsizing process rather than the genome duplication process per se that predominantly shapes the adaptive changes of polyploid lineages [118, 119, 120]. Furthermore, modelling analysis has revealed that the number of ancient polyploidy events experienced in the evolutionary past of a lineage is significantly correlated with its extant species richness [113], suggesting the additive/multiplicative effect of the ratchet of such expansion‐followed‐by‐reduction processes on lineage diversity. Nonetheless, to exert the long‐term advantage of boiling down genome redundancy, polyploids must first survive and then outcompete their diploid progenitors—a transition that rarely succeeds despite the high polyploid frequency of extant species [121], except during periods of great environmental upheaval [112]. As such, one may expect that the wave of reductive evolution is punctuated by major climatic or geological cycles such as glaciation.

### 1.4 Conclusion: Trait Volatility Born out of Repeated Shrinkage and Expansion of Coding Capacity

Genome evolution is a dynamic process; the evolutionary history of Archaeplastida is not a simple trajectory of increasing genomic and phenotypic complexity. Rather, the examples discussed here reveal a recurrent dynamic in which episodes of genomic expansion are followed by extensive and often lineage‐specific reduction, with each cycle reshaping the developmental, metabolic, and ecological potential of descendant lineages (Figure 1A,B). Red algae demonstrate that substantial ancestral gene loss need not constrain subsequent diversification but can instead be followed by the independent expansion and functional innovation of retained gene families. The zygnematophytes similarly illustrate how repeated loss and secondary innovation can generate strikingly different solutions to environmental and developmental challenges, even among lineages descending from a comparatively complex ancestor. Within embryophytes, the repeated reduction of gametophytes, stomatal toolkits, and post‐polyploid genomes further demonstrates that evolutionary success may depend not only on acquiring new genetic material, but also on selectively discarding redundancy and capacities that have become dispensable. Genome reduction therefore does not represent a unidirectional loss of evolutionary potential: it can alter the developmental and ecological landscape upon which subsequent innovation acts. Importantly, once ancestral functions have been lost, their re‐emergence may require convergent recruitment of alternative genes, horizontal transfer, or the repurposing and expansion of retained genetic components, thereby generating lineage‐specific solutions rather than restoring ancestral states. We propose that this recurrent interplay between expansion and reduction provides a useful framework for understanding trait volatility across Archaeplastida. Gene loss should consequently be considered not merely as an endpoint of genome evolution, but as an active force that continually remodels the evolutionary space from which new forms, functions, and ecological strategies emerge.

## 2 Outstanding Questions

 

− How Often After Shrinkage Do the Same Gene Families Expand Again—a Type of Parallel Evolution That Leads to the Same Recomplexification in Traits?
− Does this also happen not only if one of the gene homologs are present, but a convergent evolution of traits necessary to survive in the same niche—previously lost and subsequently innovated (or gained via HGT) again.
− Is There a Balance Between Reduction Events and Lineage Specific HGTs to Explain “Volatility”?
− Are Reduction/Loss Events the Main Drivers of Trait Volatility, or Is Lineage‐specific HGT More Important?
− Is Repeated Reductive Evolution Punctuated by Major Genome Expansion a Periodic Pattern to the Evolution of Eukaryotic and Prokaryotic Life?
− Can Neutral Processes Generate the Pattern of Repeated Reductive Evolution?
− To which Degree Can the Repeated Reductive Evolution be Gradual or Radical?
− Why Does the Opposite Pattern of Repeated Expansion Punctuated by Major Reduction Not Prevail?

## Author Contributions

**Cäcilia F. Kunz**: conceptualization, investigation, writing – original draft, writing – review and editing, funding acquisition. **Merve Nida Baştürk**: conceptualization, investigation, writing – original draft, writing – review and editing. **Jan de Vries**: conceptualization, investigation, writing – original draft, writing – review and editing, funding acquisition. **Hengchi Chen**: investigation, conceptualization, writing – original draft, writing – review and editing, funding acquisition.

## Conflicts of Interest

The authors declare no conflicts of interest.

## Data Availability

The data that support the findings of this study are available from the corresponding author upon reasonable request.

## References

[1] J. P. McCutcheon and N. A. Moran , “Extreme Genome Reduction in Symbiotic Bacteria,” Nature Reviews Microbiology 10 (2011): 13–26, 10.1038/nrmicro2670. 22064560

[2] J. P. McCutcheon , “The Genomics and Cell Biology of Host‐Beneficial Intracellular Infections,” Annual Review of Cell and Developmental Biology 37 (2021): 115–142, 10.1146/annurev-cellbio-120219-024122. 34242059

[3] A. Schoenle , O. Francis , J. M. Archibald , et al., “Protist Genomics: Key to Understanding Eukaryotic Evolution,” Trends in Genetics 41 (2025): 868–882, 10.1016/j.tig.2025.05.004. 40517085

[4] W. F. Doolittle , Y. Boucher , C. L. Nesbo , C. J. Douady , J. O. Andersson , and A. J. Roger , “How Big Is the Iceberg of which Organellar Genes in Nuclear Genomes Are but the Tip?,” Philosophical Transactions of the Royal Society of London Series B: Biological Sciences 358 (2003): 39–58, 10.1098/rstb.2002.1185. PMC1693099 12594917

[5] E. V. Koonin , “Origin of Eukaryotes From Within Archaea, Archaeal Eukaryome and Bursts of Gene Gain: Eukaryogenesis Just Made Easier?,” Philosophical Transactions of the Royal Society B: Biological Sciences 370 (2015): 20140333, 10.1098/rstb.2014.0333. PMC4571572 26323764

[6] G. Schonknecht , A. P. Weber , and M. J. Lercher , “Horizontal Gene Acquisitions by Eukaryotes as Drivers of Adaptive Evolution,” BioEssays 36 (2014): 9–20, 10.1002/bies.201300095. 24323918

[7] J. Vosseberg , J. J. E. van Hooff , M. Marcet‐Houben , et al., “Timing the Origin of Eukaryotic Cellular Complexity With Ancient Duplications,” Nature Ecology & Evolution 5 (2021): 92–100, 10.1038/s41559-020-01320-z. PMC7610411 33106602

[8] J. Van Etten and D. Bhattacharya , “Horizontal Gene Transfer in Eukaryotes: Not If, but How Much?,” Trends in Genetics 36 (2020): 915–925, 10.1016/j.tig.2020.08.006. 33012528

[9] J. P. Demuth and M. W. Hahn , “The Life and Death of Gene Families,” BioEssays 31 (2009): 29–39, 10.1002/bies.080085. 19153999

[10] M. N. Baştürk , C. F. Kunz , T. Darienko , T. Proschold , J. de Vries , and H. Chen , “Phylogenomic Frameworks From Deep Archaeplastid Evolution to Embryophyte Diversification,” Current Biology 36 (2026): R522–R544, 10.1016/j.cub.2026.03.019. 42259268

[11] W. Martin , T. Rujan , E. Richly , et al., “Evolutionary Analysis of Arabidopsis, Cyanobacterial, and Chloroplast Genomes Reveals Plastid Phylogeny and Thousands of Cyanobacterial Genes in the Nucleus,” Proceedings of the National Academy of Sciences 99 (2002): 12246–12251, 10.1073/pnas.182432999. PMC129430 12218172

[12] C. Ku , S. Nelson‐Sathi , M. Roettger , S. Garg , E. Hazkani‐Covo , and W. F. Martin , “Endosymbiotic Gene Transfer From Prokaryotic Pangenomes: Inherited Chimerism in Eukaryotes,” Proceedings of the National Academy of Sciences 112 (2015): 10139–10146, 10.1073/pnas.1421385112. PMC4547308 25733873

[13] D. Bhattacharya , H. Qiu , J. Lee , H. Su Yoon , A. P. M. Weber , and D. C. Price , “When Less Is More: Red Algae as Models for Studying Gene Loss and Genome Evolution in Eukaryotes,” Critical Reviews in Plant Sciences 37 (2018): 81–99, 10.1080/07352689.2018.1482364.

[14] J. N. Timmis , M. A. Ayliffe , C. Y. Huang , and W. Martin , “Endosymbiotic Gene Transfer: Organelle Genomes Forge Eukaryotic Chromosomes,” Nature Reviews Genetics 5 (2004): 123–135, 10.1038/nrg1271. 14735123

[15] B. J. Harris , J. W. Clark , D. Schrempf , et al., “Divergent Evolutionary Trajectories of Bryophytes and Tracheophytes From a Complex Common Ancestor of Land Plants,” Nature Ecology & Evolution 6 (2022): 1634–1643, 10.1038/s41559-022-01885-x. PMC9630106 36175544

[16] L. Guo , L. Yin , C. Sun , et al., “Gradual Genomic Streamlining and Convergent Adaptation During Terrestrial‐to‐aquatic Transitions in Angiosperms,” Current Biology 35 (2025): e4594, 10.1016/j.cub.2025.08.001. 40882631

[17] C. Guijarro‐Clarke , P. W. H. Holland , and J. Paps , “Widespread Patterns of Gene Loss in the Evolution of the Animal kingdom,” Nature Ecology & Evolution 4 (2020): 519–523, 10.1038/s41559-020-1129-2. 32094540

[18] Z. Merenyi , K. Krizsan , N. Sahu , et al., “Genomes of Fungi and Relatives Reveal Delayed Loss of Ancestral Gene Families and Evolution of Key Fungal Traits,” Nature Ecology & Evolution 7 (2023): 1221–1231, 10.1038/s41559-023-02095-9. PMC10406608 37349567

[19] M. E. Schön , V. V. Zlatogursky , R. P. Singh , et al., “Single Cell Genomics Reveals Plastid‐Lacking Picozoa are Close Relatives of Red Algae,” Nature Communications 12 (2021): 6651, 10.1038/s41467-021-26918-0. PMC8599508 34789758

[20] T. M. Gibson , P. M. Shih , V. M. Cumming , et al., “Precise Age of Bangiomorpha pubescens Dates the Origin of Eukaryotic Photosynthesis,” Geology 46 (2017): 135–138, 10.1130/g39829.1.

[21] M. D. Guiry , “Four Kingdoms, 14 Phyla, 63 Classes and Still Growing,” Journal of Phycology 48 (2012): 1057–1063, 10.1111/jpy.13431. 38245909

[22] H. S. Yoon , K. M. Müller , R. G. Sheath , F. D. Ott , and D. Bhattacharya , “Defining the Major Lineages of Red Algae (Rhodophyta),” Journal of Phycology 42 (2006): 482–492, 10.1111/j.1529-8817.2006.00210.x.

[23] G. W. Saunders and M. H. Hommersand , “Assessing Red Algal Supraordinal Diversity and Taxonomy in the Context of Contemporary Systematic Data,” American Journal of Botany 91 (2004): 1494–1507, 10.3732/ajb.91.10.1494. 21652305

[24] H. Qiu , D. C. Price , E. C. Yang , H. S. Yoon , and D. Bhattacharya , “Evidence of Ancient Genome Reduction in Red Algae (Rhodophyta),” Journal of Phycology 51 (2015): 624–636, 10.1111/jpy.12294. 26986787

[25] M. Borg , S. A. Krueger‐Hadfield , C. Destombe , J. Collen , A. Lipinska , and S. M. Coelho , “Red Macroalgae in the Genomic Era,” New Phytologist 240 (2023): 471–488, 10.1111/nph.19211. 37649301

[26] M. V. Olson , “When Less Is More: Gene Loss as an Engine of Evolutionary Change,” The American Journal of Human Genetics 64 (1999): 18–23, 10.1086/302219. PMC1377697 9915938

[27] Y. I. Wolf and E. V. Koonin , “Genome Reduction as the Dominant Mode of Evolution,” BioEssays 35 (2013): 829–837, 10.1002/bies.201300037. PMC3840695 23801028

[28] J. Lee , E. C. Yang , L. Graf , et al., “Analysis of the Draft Genome of the Red Seaweed Gracilariopsis Chorda Provides Insights Into Genome Size Evolution in Rhodophyta,” Molecular Biology and Evolution 35 (2018): 1869–1886, 10.1093/molbev/msy081. 29688518

[29] T. Lefébure , C. Morvan , F. Malard , et al., “Less Effective Selection Leads to Larger Genomes,” Genome Research 27 (2017): 1016–1028, 10.1101/gr.212589.116. PMC5453316 28424354

[30] N. J. Butterfield , “Bangiomorpha pubescensn. Gen., n. sp.: Implications for the Evolution of Sex, Multicellularity, and the Mesoproterozoic/Neoproterozoic Radiation of Eukaryotes,” Paleobiology 26 (2000): 386–404, 10.1666/0094-8373(2000)026.

[31] K. Mikami and M. Takahashi , “Life Cycle and Reproduction Dynamics of Bangiales in Response to Environmental Stresses,” Seminars in Cell & Developmental Biology 134 (2023): 14–26, 10.1016/j.semcdb.2022.04.004. 35428563

[32] K. Mikami , C. Li , R. Irie , and Y. Hama , “A Unique Life Cycle Transition in the Red Seaweed Pyropia yezoensis Depends on Apospory,” Communications Biology 2 (2019): 299, 10.1038/s42003-019-0549-5. PMC6685973 31396579

[33] J. Lee , D. Kim , D. Bhattacharya , and H. S. Yoon , “Expansion of Phycobilisome Linker Gene Families in Mesophilic Red Algae,” Nature Communications 10 (2019): 4823, 10.1038/s41467-019-12779-1. PMC6811547 31645564

[34] R. Petroll , M. Schreiber , H. Finke , J. M. Cock , S. B. Gould , and S. A. Rensing , “Signatures of Transcription Factor Evolution and the Secondary Gain of Red Algae Complexity,” Genes 12 (2021): 1055, 10.3390/genes12071055. PMC8304369 34356071

[35] R. Petroll , J. A. West , M. Ogden , et al., “The Expanded Bostrychia Moritziana Genome Unveils Evolution in the Most Diverse and Complex Order of Red Algae,” Current Biology 35 (2025): 2771–2788.e8, 10.1016/j.cub.2025.04.044. 40345196

[36] H. Qiu , J. M. Lee , H. S. Yoon , and D. Bhattacharya , “Hypothesis: Gene‐Rich Plastid Genomes in Red Algae May Be an Outcome of Nuclear Genome Reduction,” Journal of Phycology 53 (2017): 715–719, 10.1111/jpy.12514. 28095611

[37] S. Wodniok , H. Brinkmann , G. Glockner , et al., “Origin of Land Plants: Do Conjugating Green Algae Hold the Key?,” BMC Evolutionary Biology 11 (2011): 104, 10.1186/1471-2148-11-104. PMC3088898 21501468

[38] N. J. Wickett , S. Mirarab , N. Nguyen , et al., “Phylotranscriptomic Analysis of the Origin and Early Diversification of Land Plants,” Proceedings of the National Academy of Sciences 111 (2014): E4859–4868, 10.1073/pnas.1323926111. PMC4234587 25355905

[39] One Thousand Plant Transcriptomes , “One Thousand Plant Transcriptomes and the Phylogenomics of Green Plants,” Nature 574, (2019): 679–685, 10.1038/s41586-019-1693-2. PMC6872490 31645766

[40] S. Hess , S. K. Williams , A. Busch , et al., “A Phylogenomically Informed Five‐order System for the Closest Relatives of Land Plants,” Current Biology 32 (2022): 4473–4482.e7, 10.1016/j.cub.2022.08.022. PMC9632326 36055238

[41] M. J. Bierenbroodspot , T. Darienko , S. de Vries , et al., “Phylogenomic Insights Into the First Multicellular Streptophyte,” Current Biology 34 (2024): 670–681.e7, 10.1016/j.cub.2023.12.070. PMC10849092 38244543

[42] M. J. Bierenbroodspot , T. Pröschold , J. M. R. Fürst‐Jansen , et al., “Phylogeny and Evolution of Streptophyte Algae,” Annals of Botany (2024): 385–400, 10.1093/aob/mcae091. PMC11341676 38832756

[43] M. J. Bierenbroodspot , T. Darienko , S. de Vries , et al., “Phylogenomics Unveil a Recent Origin of Morphological Complexity in Coleochaetophyceae,” Current Biology 35 (2025): 5071–5080.e7, 10.1016/j.cub.2025.08.046. 40967205

[44] M. J. Bierenbroodspot , C. F. Kunz , E. S. Goldbecker , et al., “A Deep Genetic Structure Phylogenomically Frames the Closest Algal Relatives of Land Plants,” PLoS Biol 24, no. 11 (2026): e3004005, 10.1371/journal.pbio.3004005.

[45] T. Darienko , C. F. Kunz , E. S. Goldbecker , et al., “The Evolutionary Origins of Streptophyte Multicellularity,” New Phytologist (2026): 2365–2383, 10.1111/nph.71415. PMC13443282 42426897

[46] J. M. S. Zegers , L. Pfeifer , T. Darienko , et al., “Systems Acclimation to Osmotic Stress in Zygnematophyte Cells,” Nature Communications (2026): 755, 10.1038/s41467-026-68329-z. PMC12820192 41545391

[47] X. Feng , J. Zheng , I. Irisarri , et al., “Genomes of Multicellular Algal Sisters to Land Plants Illuminate Signaling Network Evolution,” Nature Genetics 56 (2024): 1018–1031, 10.1038/s41588-024-01737-3. PMC11096116 38693345

[48] E. S. Goldbecker , D. Varshney , A. Holzhausen , et al., “The *Spirogyra pratensis* Genome Illuminates the Evolution of Developmental Programs and Spiral Chloroplast Biology,” Plant Cell 38 (2026): koag224, 10.1093/plcell/koag224. PMC13519894 42520407

[49] C. Jiao , I. Sørensen , X. Sun , et al., “The Penium Margaritaceum Genome: Hallmarks of the Origins of Land Plants,” Cell 181 (2020): 1097–1111.e12, 10.1016/j.cell.2020.04.019. 32442406

[50] L. A. Lewis and R. M. McCourt , “Green Algae and the Origin of Land Plants,” American Journal of Botany 91 (2004): 1535–1556, 10.3732/ajb.91.10.1535. 21652308

[51] R. M. McCourt , L. A. Lewis , P. K. Strother , et al., “Green Land: Multiple Perspectives on Green Algal Evolution and the Earliest Land Plants,” American Journal of Botany 110 (2023): e16175, 10.1002/ajb2.16175. 37247371

[52] J. Ma , S. Wang , X. Zhu , et al., “Major Episodes of Horizontal Gene Transfer Drove the Evolution of Land Plants,” Molecular Plant 15 (2022): 857–871, 10.1016/j.molp.2022.02.001. 35235827

[53] J. W. Clark , “Genome Evolution in Plants and the Origins of Innovation,” New Phytologist 240 (2023): 2204–2209, 10.1111/nph.19242. 37658677

[54] A. M. Linde , D. M. Eklund , A. Kubota , et al., “Early Evolution of the Land Plant Circadian Clock,” New Phytologist 216 (2017): 576–590, 10.1111/nph.14487. PMC5638080 28244104

[55] T. Vogt , “Phenylpropanoid Biosynthesis,” Molecular Plant 3 (2010): 2–20, 10.1093/mp/ssp106. 20035037

[56] R. A. Dixon and J. Barros , “Lignin Biosynthesis: Old Roads Revisited and New Roads Explored,” Open Biology 9 (2019): 190215, 10.1098/rsob.190215. PMC6936255 31795915

[57] R. Vanholme , B. De Meester , J. Ralph , and W. Boerjan , “Lignin Biosynthesis and Its Integration Into Metabolism,” Current Opinion in Biotechnology 56 (2019): 230–239, 10.1016/j.copbio.2019.02.018. 30913460

[58] S. de Vries , J. M. R. Fürst‐Jansen , I. Irisarri , et al., “The Evolution of the Phenylpropanoid Pathway Entailed Pronounced Radiations and Divergences of Enzyme Families,” The Plant Journal 107 (2021): 975–1002, 10.1111/tpj.15387. 34165823

[59] L. Labeeuw , P. T. Martone , Y. Boucher , and R. J. Case , “Ancient Origin of the Biosynthesis of Lignin Precursors,” Biology Direct 10 (2015): 23, 10.1186/s13062-015-0052-y. PMC4455696 25994183

[60] J. de Vries , S. de Vries , C. H. Slamovits , L. E. Rose , and J. M. Archibald , “How Embryophytic Is the Biosynthesis of Phenylpropanoids and Their Derivatives in Streptophyte Algae?,” Plant & Cell Physiology 58 (2017): 934–945, 10.1093/pcp/pcx037. 28340089

[61] C. M. Schwarze and M. Petersen , “Phenylalanine Ammonia‐Lyases and 4‐Coumaric Acid Coenzyme a Ligases in Chara braunii, Marchantia polymorpha, and Physcomitrium patens As Extant Model Organisms for Plant Terrestrialization,” The Plant Journal (2024): 2797–2815, 10.1111/tpj.16950. 39052447

[62] R. Stracke , J. J. Favory , H. Gruber , et al., “The Arabidopsis bZIP Transcription Factor HY5 Regulates Expression of the PFG1/MYB12 Gene in Response to Light and Ultraviolet‐B Radiation,” Plant, Cell and Environment 33 (2010): 88–103, 10.1111/j.1365-3040.2009.02061.x. 19895401

[63] S. Takshak and S. B. Agrawal , “Secondary Metabolites and Phenylpropanoid Pathway Enzymes as Influenced Under Supplemental Ultraviolet‐B Radiation in Withania Somnifera Dunal, an Indigenous Medicinal Plant,” Journal of Photochemistry and Photobiology B: Biology 140 (2014): 332–343, 10.1016/j.jphotobiol.2014.08.011. 25226342

[64] Y. Song , B. Ma , Q. Guo , et al., “UV‐B Induces the Expression of Flavonoid Biosynthetic Pathways in Blueberry (Vaccinium corymbosum) Calli,” Frontiers in Plant Science 13 (2022): 1079087, 10.3389/fpls.2022.1079087. PMC9722975 36483950

[65] C. F. Kunz , I. N. Abreu , T. Darienko , et al., “Chemodiverse Cell System Responses to UV in an Algal Sister of Land Plants,” Current Biology (2026): 2673–2687.e6, 10.1016/j.cub.2026.04.015. 42092354

[66] D. Remias , S. Schwaiger , S. Aigner , T. Leya , H. Stuppner , and C. Lutz , “Characterization of an UV‐ and VIS‐absorbing, Purpurogallin‐derived Secondary Pigment New to Algae and Highly Abundant in Mesotaenium Berggrenii (Zygnematophyceae, Chlorophyta), an Extremophyte Living on Glaciers,” FEMS Microbiology Ecology 79 (2012): 638–648, 10.1111/j.1574-6941.2011.01245.x. 22092588

[67] A. M. C. Bowles , T. A. Williams , P. C. Donoghue , D. A. Campbell , and C. J. Williamson , “Metagenome‐Assembled Genome of the Glacier Alga Ancylonema Yields Insights Into the Evolution of Streptophyte Life on Ice and Land” New Phytologist 244 (2024): 1125–1127. 10.1111/nph.19860 38840553

[68] C. F. Kunz , S. de Vries , and J. Vries , “Plant Terrestrialization: An Environmental Pull on the Evolution of Multi‐sourced Streptophyte Phenolics,” Philosophical Transactions of the Royal Society B: Biological Sciences 379 (2024): 20230358, 10.1098/rstb.2023.0358. PMC11528360 39343031

[69] C. F. Kunz , E. S. Goldbecker , T. Darienko , and J. Vries , “Genome Evolution: Zygnematophyceae on Ice,” New Phytologist 244 (2024): 1125–1127, 10.1111/nph.19960. 39001590

[70] A. Busch and S. Hess , “Sunscreen Mucilage: A Photoprotective Adaptation Found in Terrestrial Green Algae (Zygnematophyceae),” European Journal of Phycology 57 (2021): 107–124, 10.1080/09670262.2021.1898677.

[71] A. Busch , J. V. Gerbracht , K. Davies , U. Hoecker , and S. Hess , “Comparative Transcriptomics Illuminates the Cellular Responses of an Aeroterrestrial Zygnematophyte to UV Radiation,” Journal Experimental Botany 75 (2024): 3624–3642, 10.1093/jxb/erae131. PMC11156808 38520340

[72] M. Pichrtová , D. Remias , L. A. Lewis , and A. Holzinger , “Changes in Phenolic Compounds and Cellular Ultrastructure of Arctic and Antarctic Strains of Zygnema (Zygnematophyceae, Streptophyta) After Exposure to Experimentally Enhanced UV to PAR Ratio,” Microbial ecology 65 (2013): 68–83, 10.1007/s00248-012-0096-9. PMC3541927 22903087

[73] L. Procházková , P. Mojzes , J. Racek , L. Nedbalova , and D. Remias , “Phenolic Iron Complexes Protect Glacier Ice Algae (Zygnematophyceae) against Excessive UV and VIS Irradiation,” Environmental Microbiology Reports 17 (2025): e70149, 10.1111/1758-2229.70149. PMC12257149 40654230

[74] L. Hoffmann , “Algae of Terrestrial Habitats,” The Botanical Review 55 (1989): 77–105, 10.1007/bf02858529.

[75] J. de Vries and J. M. Archibald , “Plant Evolution: Landmarks on the Path to Terrestrial Life,” New Phytologist 217 (2018): 1428–1434, 10.1111/nph.14975. 29318635

[76] P. C. J. Donoghue , C. J. Harrison , J. Paps , and H. Schneider , “The Evolutionary Emergence of Land Plants,” Current Biology 31 (2021): R1281–R1298, 10.1016/j.cub.2021.07.038. 34637740

[77] E. S. Goldbecker and J. Vries , “Systems Biology of Streptophyte Cell Evolution,” Annual Review of Plant Biology 76 (2025): 493–522, 10.1146/annurev-arplant-083123-060254. 39819561

[78] C. F. Kunz , E. S. Goldbecker , and J. Vries , “Functional Genomic Perspectives on Plant Terrestrialization,” Trends in Genetics 41 (2025): 617–629, 10.1016/j.tig.2025.02.006. 40155238

[79] B. Kenrick , “Alternation of Generations in Land Plants: New Phylogenetic and Palaeobotanical Evidence,” Biological Reviews 69 (2008): 293–330, 10.1111/j.1469-185X.1994.tb01273.x.

[80] E. Dominguez , J. A. Heredia‐Guerrero , and A. Heredia , “The Plant Cuticle: Old Challenges, New Perspectives,” Journal of Experimental Botany 68 (2017): 5251–5255, 10.1093/jxb/erx389. PMC5853762 29136457

[81] B. J. Harris , C. J. Harrison , A. M. Hetherington , and T. A. Williams , “Phylogenomic Evidence for the Monophyly of Bryophytes and the Reductive Evolution of Stomata,” Current Biology 30 (2020): 2001–2012.e2, 10.1016/j.cub.2020.03.048. 32302587

[82] J. M. R. Fürst‐Jansen , S. de Vries , and J. Vries , “Evo‐physio: On Stress Responses and the Earliest Land Plants,” Journal of Experimental Botany 71 (2020): 3254–3269, 10.1093/jxb/eraa007. PMC7289718 31922568

[83] J. Harrison , “Development and Genetics in the Evolution of Land Plant Body Plans,” Philosophical Transactions of the Royal Society of London Series B: Biological Sciences 372 (2017): 20150490, 10.1098/rstb.2015.0490. PMC5182422 27994131

[84] N. D. Pires and L. Dolan , “Morphological Evolution in Land Plants: New Designs With Old Genes,” Philosophical Transactions of the Royal Society B: Biological Sciences 367 (2012): 508–518, 10.1098/rstb.2011.0252. PMC3248709 22232763

[85] Z. Xu , H. Wang , Z. Zhang , and B. Zhong , “Adaptive Evolutionary Shifts in De Novo Birth and Fixation Strategy of Lineage‐Specific Genes During Plant Terrestrialization,” The Plant Journal 126 (2026): e70977, 10.1111/tpj.70977. 42251754

[86] E. S. Sorojsrisom , D. W. Stevenson , and B. A. Ambrose , “Patterning and Evolution of the Land Plant Gametangia,” International Journal of Plant Sciences 187 (2026): 1–70, 10.1086/737170.

[87] P. Gerrienne and P. Gonez , “Early Evolution of Life Cycles in Embryophytes: A Focus on the Fossil Evidence of Gametophyte/Sporophyte Size and Morphological Complexity,” Journal of Systematics and Evolution 49 (2011): 1–16, 10.1111/j.1759-6831.2010.00096.x.

[88] K. J. Niklas and U. Kutschera , “The Evolution of the Land Plant Life Cycle,” New Phytologist 185 (2010): 27–41, 10.1111/j.1469-8137.2009.03054.x. 19863728

[89] A. H. Knoll , K. J. Niklas , P. G. Gensel , and B. H. Tiffney , “Character Diversification and Patterns of Evolution in Early Vascular Plants,” Paleobiology 10 (2016): 34–47, 10.1017/s0094837300008009.

[90] R. M. Bateman and W. A. DiMichele , “Heterospory: The Most Iterative Key Innovation in the Evolutionary History of the Plant Kingdom,” Biological Reviews 69 (2008): 345–417, 10.1111/j.1469-185X.1994.tb01276.x.

[91] C. H. Haufler , K. M. Pryer , E. Schuettpelz , et al., “Sex and the Single Gametophyte: Revising the Homosporous Vascular Plant Life Cycle in Light of Contemporary Research,” Bioscience 66 (2016): 928–937, 10.1093/biosci/biw108.

[92] X. Wang and S. N. Bai , “Key Innovations in Transition From Homospory to Heterospory,” Plant Signaling & Behavior 14 (2019): 1596010, 10.1080/15592324.2019.1596010. PMC6546143 30892985

[93] I. J. Leitch and A. R. Leitch , “Genome Size Diversity and Evolution in Land Plants,” in Plant Genome Diversity Volume 2: Physical Structure, Behaviour and Evolution of Plant Genomes, ed. J. Greilhuber , J. Dolezel , and J. F. Wendel , (Springer Vienna, 2013), 307–322, 10.1007/978-3-7091-1160-4_19.

[94] P. Bures , K. Vejvodova , F. Zedek , et al., “Divergent Trajectories of Genome Architecture and Chromosome Evolution in Ferns and Angiosperms,” New Phytolpgy 252 (2026): 427–442, 10.1111/nph.71458. PMC13539971 42504640

[95] S. P. Kinosian and M. S. Barker , “The Role of Meiotic Drive in Chromosome Number Disparity between Heterosporous and Homosporous Plants,” Molecular Ecology 34 (2025): e17757, 10.1111/mec.17757. 40192417

[96] S. P. Kinosian , “Genomics Is Modifying Our Concepts of Evolution in Homosporous and Heterosporous Vascular Plants,” American Journal of Botany (2026): e70248, 10.1002/ajb2.70248. PMC13496105 42579506

[97] K. Plackova , P. Bures , M. A. Lysak , and F. Zedek , “Centromere Drive May Propel the Evolution of Chromosome and Genome Size in Plants,” Annals of Botany 134 (2024): 1067–1076, 10.1093/aob/mcae149. PMC11687628 39196767

[98] D. C. Presgraves , R. K. Dawe , K. A. Dyer , et al., “The Evolutionary Genomics of Meiotic Drive,” Molecular Biology and Evolution 43 (2026): msag020, 10.1093/molbev/msag020. PMC12941216 41589062

[99] J. W. Clark , B. J. Harris , A. J. Hetherington , et al., “The Origin and Evolution of Stomata,” Current Biology 32 (2022): R539–R553, 10.1016/j.cub.2022.04.040. 35671732

[100] A. M. Hetherington and F. I. Woodward , “The Role of Stomata in Sensing and Driving Environmental Change,” Nature 424 (2003): 901–908, 10.1038/nature01843. 12931178

[101] S. M. Assmann and T. Jegla , “Guard Cell Sensory Systems: Recent Insights on Stomatal Responses to Light, Abscisic Acid, and CO_2_ ,” Current Opinion in Plant Biology 33 (2016): 157–167, 10.1016/j.pbi.2016.07.003. 27518594

[102] T. Lawson and J. Matthews , “Guard Cell Metabolism and Stomatal Function,” Annual Review of Plant Biology 71 (2020): 273–302, 10.1146/annurev-arplant-050718-100251. 32155341

[103] J. de Vries , B. A. Curtis , S. B. Gould , and J. M. Archibald , “Embryophyte Stress Signaling Evolved in the Algal Progenitors of Land Plants,” PNAS 115 (2018): E3471–E3480, 10.1073/pnas.1719230115. PMC5899452 29581286

[104] Y. Sun , B. Harpazi , A. Wijerathna‐Yapa , et al., “A Ligand‐Independent Origin of Abscisic Acid Perception,” Proceedings of the National Academy of Sciences 116 (2019): 24892–24899, 10.1073/pnas.1914480116. PMC6900503 31744875

[105] G. Zimran , M. Shpilman , E. Hobson , et al., “Abscisic Acid Receptors Functionally Converge Across 500 Million Years of Land Plant Evolution,” Current Biology 35 (2025): e814, 10.1016/j.cub.2024.12.043. 39892384

[106] J. M. S. Zegers , G. X. Garcia Ramirez , A. Harzen , et al., “Homologous ABA‐Independent Kinase Tracks Coalesced Into Osmotic Stress Circuits During Plant Terrestrialization,” bioRxiv (2026), 10.64898/2026.05.21.726866.

[107] K. S. Renzaglia , W. B. Browning , and A. Merced , “With Over 60 Independent Losses, Stomata are Expendable in Mosses,” Frontiers in Plant Science 11 (2020): 567, 10.3389/fpls.2020.00567. PMC7270291 32547571

[108] K. S. Renzaglia , J. C. Villarreal , B. T. Piatkowski , J. R. Lucas , and A. Merced , “Hornwort Stomata: Architecture and Fate Shared With 400‐Million‐Year‐Old Fossil Plants Without Leaves,” Plant Physiology 174 (2017): 788–797, 10.1104/pp.17.00156. PMC5462037 28584065

[109] X. Ma , S. Vanneste , J. Chang , et al., “Seagrass Genomes Reveal Ancient Polyploidy and Adaptations to the Marine Environment,” Nature Plants 10 (2024): 240–255, 10.1038/s41477-023-01608-5. PMC7615686 38278954

[110] J. G. Duckett , K. S. Renzaglia , and S. Pressel , “Reflections on the Absence of Stomata on the Gametophyte Generation of Extant Land Plants: A Focus on Poikilohydry,” Botanical Journal of the Linnean Society 208 (2025): 14–28, 10.1093/botlinnean/boae055.

[111] S. Mursidawati , A. Wicaksono , and T. Silva , “Rafflesia Patma Blume Flower Organs: Histology of the Epidermis and Vascular Structures, and a Search for Stomata,” Planta 251 (2020): 112, 10.1007/s00425-020-03402-5. 32494866

[112] H. Chen , F. Almeida‐Silva , G. Logghe , S. Maere , D. Bonte , and Y. Van de Peer , “The Rise of Polyploids During Environmental Upheaval,” Cell 189 (2026): 3608–3622.e5, 10.1016/j.cell.2026.04.008. 42105759

[113] Z. Ali , Q. W. Tan , P. K. Lim , et al., “Comparative Transcriptomics in Ferns Reveals Key Innovations and Divergent Evolution of the Secondary Cell Walls,” Nature Plants 11 (2025): 1028–1048, 10.1038/s41477-025-01978-y. 40269175

[114] S. Dong , S. Wang , L. Li , et al., “Bryophytes Hold a Larger Gene family Space Than Vascular Plants,” Nature Genetics 57 (2025): 2562–2569, 10.1038/s41588-025-02325-9. PMC12513842 40983756

[115] S.‐Z. Lin , Y. Chen , C. Wu , et al., “Cunninghamia lanceolata Genome Illuminates the Evolutionary Dynamics of Gymnosperms,” Cell Reports 45 (2026): 117566, 10.1016/j.celrep.2026.117566. PMC13415678 42319826

[116] D. Bonte and Y. Van de Peer , “A Three‐step Model for the Establishment of Polyploid Plants,” Current Biology 36 (2026): R553–R564, 10.1016/j.cub.2026.03.078. 42259270

[117] J. B. Landis , D. E. Soltis , Z. Li , et al., “Impact of Whole‐Genome Duplication Events on Diversification Rates in Angiosperms,” American Journal of Botany 105 (2018): 348–363, 10.1002/ajb2.1060. 29719043

[118] S. Grunig , R. R. Choudhury , and C. Parisod , “Evolution After Whole‐Genome Duplication (WGD) Drives Phenotypic and Transcriptomic Divergence More Than WGD in an Autopolyploid Herb,” Proceedings of the National Academy of Sciences 123 (2026): e2533430123, 10.1073/pnas.2533430123. PMC13462920 42550909

[119] S. Dodsworth , M. W. Chase , and A. R. Leitch , “Is Post‐Polyploidization Diploidization the Key to the Evolutionary Success of Angiosperms?,” Botanical Journal of the Linnean Society 180 (2016): 1–5, 10.1111/boj.12357.

[120] X. Feng , Q. Chen , W. Wu , et al., “Genomic Evidence for Rediploidization and Adaptive Evolution Following the Whole‐Genome Triplication,” Nature Communications 15 (2024): 1635, 10.1038/s41467-024-46080-7. PMC10884412 38388712

[121] A. Rice , P. Smarda , M. Novosolov , et al., “The Global Biogeography of Polyploid Plants,” Nature Ecology & Evolution 3 (2019): 265–273, 10.1038/s41559-018-0787-9. 30697006

